# Magnitude and associated factors of intraoperative cardiac complications among geriatric patients who undergo non-cardiac surgery at public hospitals in the southern region of Ethiopia: a multi-center cross-sectional study in 2022/2023

**DOI:** 10.3389/fmed.2024.1325358

**Published:** 2024-04-17

**Authors:** Amina Abdulmelik, Mebratu Tila, Takele Tekilu, Ashebir Debalkie, Elias Habtu, Ashagrie Sintayehu, Getahun Dendir, Naol Gordie, Abel Daniel, Mohammed Suleiman Obsa

**Affiliations:** ^1^School of Anesthesia, College of Health Science and Medicine, Wolaita Soddo University, Wolaita Soddo, Ethiopia; ^2^School of Medical Laboratory, College of Medicine and Health Science, Wolaita Soddo University, Wolaita Soddo, Ethiopia; ^3^School of Medicine, College of Medicine and Health Science, Wolaita Soddo University, Wolaita Soddo, Ethiopia; ^4^Department of Anesthesia, College of Medicine and Health Science, Arsi University, Assela, Ethiopia

**Keywords:** magnitude, associated factors, intraoperative, cardiac complications, geriatrics, non-cardiac surgery

## Abstract

**Background:**

Intraoperative cardiac complications are a common cause of morbidity and mortality in non-cardiac surgery. The risk of these complications increased with the average age increasing from 65. In a resource-limited setting, including our study area, the magnitude and associated factors of intraoperative cardiac complications have not been adequately investigated. The aim of this study was to assess the magnitude and associated factors of intraoperative cardiac complications among geriatric patients undergoing non-cardiac surgery.

**Methods:**

An institutional-based multi-center cross-sectional study was conducted on 304 geriatric patients at governmental hospitals in the southern region of Ethiopia, from 20 March 2022 to 25 August 2022. Data were collected by chart review and patient interviews. Epi Data version 4.6 and SPSS version 25 were used for analysis. The variables that had association (*p* < 0.25) were considered for multivariable logistic regression. A *p* value < 0.05 was considered significant for association.

**Result:**

The overall prevalence of intraoperative cardiac complications was 24.3%. Preoperative ST-segment elevation adjusted odds ratio (AOR = 2.43, CI =2.06–3.67), history of hypertension (AOR = 3.42, CI =2.02–6.08), intraoperative hypoxia (AOR = 3.5, CI = 2.07–6.23), intraoperative hypotension (AOR = 6.2 9, CI =3.51–10.94), age > 85 years (AOR = 6.01, CI = 5.12–12.21), and anesthesia time > 3 h (AOR =2.27, CI = 2.0.2–18.25) were factors significantly associated with intraoperative cardiac complications.

**Conclusion:**

The magnitude of intraoperative cardiac complications was high among geriatric patients who had undergone non-cardiac surgery. The independent risk factors of intraoperative cardiac complications for this population included age > 85, ST-segment elevation, perioperative hypertension (stage 3 with regular treatment), duration of anesthesia >3 h, intraoperative hypoxia, and intraoperative hypotension. Holistic preoperative evaluation, optimization optimal and perioperative care for preventing perioperative risk factors listed above, and knowing all possible risk factors are suggested to reduce the occurrence of complications.

## 1 Introduction

Intraoperative cardiac complication (ICC) is defined as follows: any death, unless unequivocal non-cardiac cause can be established; non-fatal cardiac arrest (absence of cardiac rhythm or the presence of chaotic rhythm requiring any component of basic or advanced cardiac life support) and at least one of the ischemic symptoms (1). Intraoperative management of geriatric surgical patients is becoming an increasingly important component of surgical and anesthetic practice in the 21st century. With an average age of 65, the risk of cardiac complications is increasing (2).

A study of more than 100,000 patients, which included geriatric and non-cardiac surgical patients, showed that at least 30% of mortality during the surgical period was caused by cardiac complications. As a result, more than 3,600 patients die each year due to cardiac complications of non-cardiac surgery in the Netherlands alone (1).

ICC definition: If any of the following ICCs are present during the intraoperative period. These include congestive heart failure, non-fatal cardiac arrest, elevation or depression of the ST segment, and cardiac death (2).

According to the World Health Organization (WHO), most developed countries have accepted the chronological age of 65 years and above as a definition of the elderly. Although there are different ways to classify this population, some studies have classified elderly adults between the ages of 65 and 74 as “youngest-old,” those between ages 75 and 84 as “middle-aged,” and those over 85 as “oldest-old (3).”

Notably, cardiovascular events are relatively frequent in non-cardiac surgery, with an estimated prevalence of 1%–5% and intraoperative infarctions cause mortality in the range of 25%–40% (3).

Major non-cardiac surgery is associated with incidence of cardiac death between 0.5% and 1.5% and major cardiac complications between 2.0% and 3.5%. When applied to the European population, these figures translate into 150,000–250,000 life-threatening cardiac complications due to non-cardiac surgical procedures annually (4). The manifestations of intraoperative cardiac complications (ICC) are usually subtle; therefore, doctors discover perioperative myocardial infarction (PMI) very late, leading to a 30%–70% mortality rate. Despite decades of research on the prediction and prevention of cardiac events, the prevalence of ICC has remained constant, risk factors in the elderly may differ significantly from those in the general population, and the prevalence of ICC remains elevated (5, 6). Major cardiac complications associated with abdominal and non-cardiac thoracic surgery are a common cause of mortality and serious morbidity in elderly patients (3, 7). The oldest age group underwent the smallest range of procedures; hip fracture, hip replacement, and cataract procedures comprised greater than 35% of all surgeries. Mortality and complications were increased in the geriatric compared with younger adults (5).

In general, elderly people face chronic illnesses that have risk of intraoperative cardiac complications. According to ICD-9 codes 375–449, cardiac death was identified as the leading cause of death as indicated on the patient’s death certificate (8, 9). A study of more than 100,000 patients, which included geriatric and non-cardiac surgical patients, showed that at least 30% of mortality during the surgical period was caused by cardiac complications. As a result, more than 3,600 patients die each year of cardiac complications of non-cardiac surgery in the Netherlands alone (10). Intraoperative mortality occurred in 1,877 (1.7%) patients and cardiovascular mortality in 543 (0.5%) cases. The study also enrolled 3,893 surgical patients, of whom 136 (3.5%) had intraoperative cardiac death. The study enrolled 8,351 patients undergoing non-cardiac surgery, Intraoperative mortality occurred in 226 patients (2.7%), of whom 133 (1.6%) suffered cardiovascular death (11).

Hypertension and diabetes are common diseases that result in cardiac morbidity and mortality. At the same time, major surgical procedures are being performed on elderly patients with increasing frequency. The risk of heart complications increases as one’s age approaches 65 years. Several studies have described the association between cardiac morbidity and mortality in patients undergoing non-cardiac surgery, but few data are available to guide preoperative risk stratification. In addition, these studies fail to address the question the factors associated with intraoperative cardiac morbidity and mortality (12).

Despite advances in patient management, morbidity and mortality secondary to ICC remain significantly high in developed countries, including the United States. In a resource-limited setting such as Ethiopia, including our study area, the magnitude and associated factors of ICC have not been adequately investigated.

Most studies on this topic did not include common associated factors that are included in this study, such as preoperative coexisting disease. Therefore, this study will fill the gap and have a positive impact on identifying associated factors that were not included in prior studies. In addition, some studies on current topics produced contradictory results and were unable to reach a consensus on the magnitude and associated factors, resulting in a longer length of stay, higher healthcare costs, and a worse prognosis. This might be a source of difficulty among health professionals in making decisions for geriatric patients undergoing non-cardiac surgery. Previous studies that were conducted on the ICC used retrospective data from a single institution with a small sample size, which was stated as a limitation in their study. To provide only preliminary data regarding intraoperative cardiac complications, this was multi-center study that used primary data. This will resolve the limitation. The findings of this study would help anesthesia professionals, physicians, and PACU nurses to know and provide optimal management during perioperative care. The aim of the current study was to assess the magnitude and associated factors of intraoperative cardiac complications among geriatric patients undergoing non-cardiac surgery.

## 2 Materials and methods

### 2.1 Study area

This study was conducted at three selected public hospitals in the Southern Nation Nationality and People Region (SNNPR), Ethiopia, from 20 March 2022 to 25 August 2022. SNNPR is one of the largest regions in Ethiopia, accounting for more than 10% of the country’s land area and with an estimated population of 20,768,000 nearly a fifth of the country’s population. Administratively, the region is divided into 14 zones, 1 city administration, and 4 special districts. According to the regional health bureau’s annual report, there are 21 governmental hospitals (13).

Wolaita Sodo University Comprehensive and Specialized Hospital (WSUCSH) was established in 1920 E. C and is located in Wolaita Sodo town, 380 km south of Addis Ababa. It has served as a comprehensive and specialized since 2021, which delivers different medical services for outpatients, emergency operation rooms (OPD rooms), gynecology and obstetrics, and inpatients for approximately 450–500 patients per day for 24 h. The total service coverage of the hospital is approximately 3 million people in catchment areas (14).

Worabe Comprehensive and Specialized Hospital (WCSH) is located in the Site found in Zone, Southern Ethiopia in Worabe Town, which is located 172 km southwest of Addis Ababa, Ethiopia’s capital. It was established in 2014. It has 200 beds. The services in the hospital include outpatient department (OPD), inpatient, operation room gynecology and obstetrics, emergency, laboratory, pharmacy, and imaging services (15). Nigist Eleni Mohammed Memorial Referral Hospital (NEMMRH), Hadiya zone, Hosanna, Southern Ethiopia is located approximately 232 km southwest of Addis Ababa. The hospital was established in 1990. The hospital is well-equipped with medical equipment and staff. It has 9 wards and a total of 220 beds, including 8 and 7 beds in neonatal and adult ICUs (intensive care), respectively. The main health services in this hospital are inpatient, operation room, gynecology and obstetrics, and emergency and critical care (16).

An institution-based multi-center cross-sectional study design was employed.

The sample of 304 participants was determined using a single population formula for a finite population with assumptions of 95% CI, marginal error (d) = 5%, and prevalence (p) = 0.26.


Q=1−p=0.74

$$n=\frac{{\left(Z\frac{a}{2}\right)}^{2}P\;\left(1-P\right)}{d^{2}}=\;\frac{{\left(1.96\right)}^{2}\;0.26\left(1-0.26\right)}{{\left(0.05\right)}^{2}}$$

$$\left(1.9208\right)\;\left(0.74\right)=295$$

Therefore, the sample size required is 295. By considering a 5% non-response rate, the total sample size is 304.

The proportional allocation of sample size was applied to hospitals using last year’s performance report for the randomly selected hospital. Here, hospitals serve as strata; the patients, wherever they appear, are similar regarding the surgical procedures being performed, the level of health professionals, and hospital facilities, so no design effect was used. Patients over 65 years of age who had an electrocardiogram performed per institutional guidelines, ASA I and ASA II, and elective surgeries were included. Vascular surgery, eye surgery intraoperative lidocaine infiltration, or more during the same hospitalization were excluded from the study.

### 2.2 Data collection tools and procedures

We pretest 5% of sample size on Hawassa Comprehensive Specialized Hospital. Two MSc students of advanced clinical anesthesia and two MSc advanced clinical anesthesia were at the end of surgery by chart reviewing using structured data collection tool. Preoperative ECGs were analyzed a using the Minnesota Codes, to determine evidence of myocardial infarction (MI) and other ECG abnormalities in patients undergoing surgery in a standardized fashion. Utilising preoperative risk factors according to Lee’s Revised Cardiac Risk Index (RCRI for Pre-Operative Risk), patients with comorbidities were predefined (17), and the patients were determined according to the number of comorbidities such as ischemic heart disease, CHF, and insulin use. Patients were deemed to have ischemic heart disease if they had a history of MI, any history of angina pectoris, or cardiac chest pain. In addition, patients with a creatinine level of >2 mg/dL will be assessed for intraoperative cardiac complications (18). Intraoperative vital signs: Blood pressure and heart rate were recorded, and 6-lead ECG was performed.

Patients were assessed for any signs and symptoms of ICC including unstable angina, acute myocardial infarction (AMI), new-onset severe arrhythmia, heart failure (HF), non-fatal cardiac arrest, cardiac death, ST-segment elevation as major criteria and symptoms of ischemia such as chest pain or dyspnea, ECG changes indicating new ischemia, new ST-T changes bundle branch block, and new pathological Q waves on ECG (19).

The patient who developed one of the major criteria and at least one symptom of ischemic was considered an intraoperative cardiac complication.

### 2.3 Data analysis

The data were entered into Epi data version 4.6 before being transferred to SPSS version 25 for analysis. Depending on the distribution, categorical variables are displayed as number frequencies and percentages. Multicollinearity was tested using the coefficients’ covariance matrix, and VIF was checked. A binary logistic regression model was used. The variables that had association (*p* < 0.25) were entered and analyzed by a multivariable logistic regression model to identify the independent effect of different factors. Adjusted odds ratio (AOR) with a 95% CI was used to identify the strength of the association. Statistical significance was declared at a *p*-value of <0.05.

### 2.4 Operational definitions

Outcome Measurement has been measured through clinical and intraoperative routine monitoring of blood pressure (BP), pulse rate, oxygen saturation (SpO2), and ECG changes.
ICC definition at least one of the following ICCs occurring intraoperative: cardiac death, non-fatal cardiac arrest, ST-segment elevation or depression; congestive heart failure (20).
Cardiac death: any death, unless an unequivocal non-cardiac cause could be established (18, 19, 21).
Adults between the ages of 65 and 74 years as the youngest-old, those between ages 75 and 84 years as middle-old, and those aged over 85 years as the oldest-old (22).

### 2.5 Ethical consideration

The institutional review board of Wolaita Sodo University, College of Health Science and Medicine, granted ethical clearance and approval under project number CHSM-ERC-03-12, reference number CRCD 82-02-14. An official letter of support and permission to conduct out the study was received from WSUCSH and other rest of the study area hospitals. Informed consent was obtained from the participants.

## 3 Results

Of 304 geriatric patients, 100% were included in the analysis. Of the total patients included in the analysis, 52.6% and 47.4% were male and female patients, respectively. Most of the patients (71.7%) were aged between 75 and 84 years, and 22 (7.2%) of the study patients were obese with a BMI of over 30. The majority of the study population (60.2%) was from rural areas, and 33.6% of the study population was from WSUSCH. The demographic characteristics of the 304 patients are shown in Table 1.

**Table 1:** Socio-demographic factors of the study population at selected public hospitals in SSNPR, Ethiopia, from March 2022 to August 2022.

Variables		Frequency	Percent (%)
Age	65–74 (youngest-old)	33	10.9
	75–84 (middle-old)	218	71.7
	>85 (oldest-old)	43	16.5
BMI (Kg/m^2^)	<18.5 (underweight)	9	3
	18.5–24.5 (normal)	201	66. 1
	25–30 (overweight)	72	23.7
	>30 (obesity)	22	7.2
Gender	Female	160	52.6
	Male	144	47.4
Address	Urban	121	39.8
	Rural	183	60.2
Hospitals	WSUCSH	102	33.6
	WCSH	102	33.6
	NEMMRH	100	32.8

BMI, Body mass index, %, percent; WSUCSH, Wolaita Sodo University Comprehensive and Specialized Hospital; WSCH, Worabe Comprehensives Hospital; NEMMRH, Nigist Eleni Mohammed Memorial Referral Hospital.

### 3.1 Magnitude of intraoperative cardiac complications

**Figure 1:**
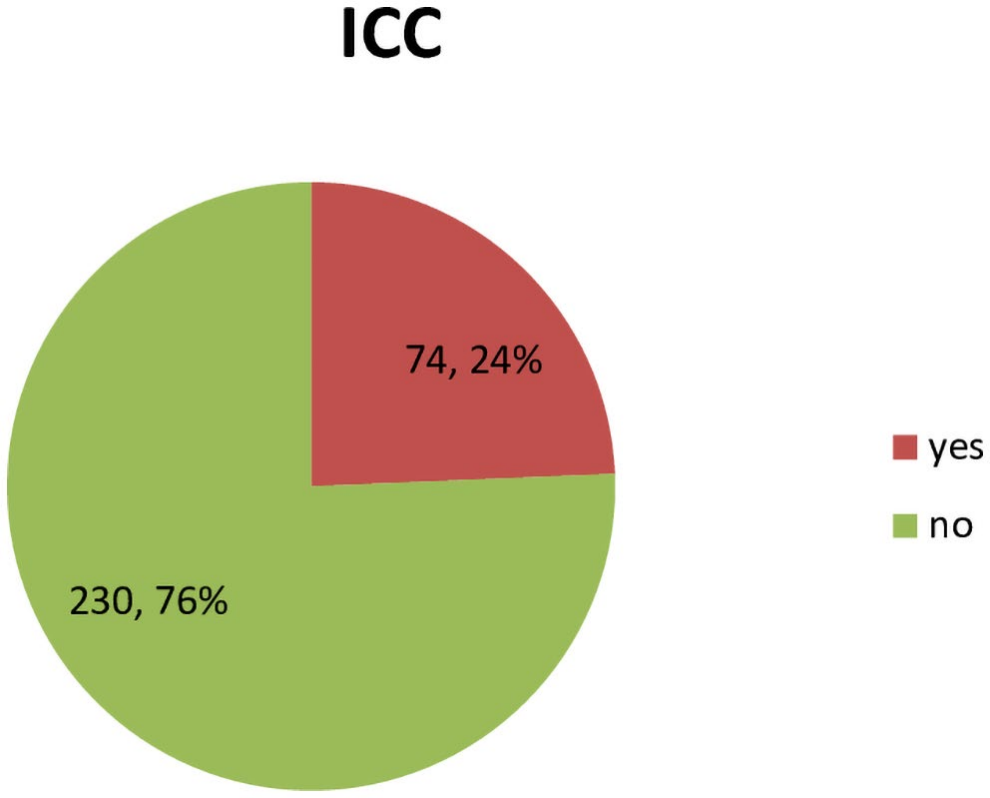
Overall magnitude of intraoperative cardiac complications at selected SNNPR governmental hospitals, from March 2022 to August 2022.

Among 304 geriatric patients undergoing elective non-cardiac surgery at selected SNNPR governmental hospitals in Ethiopia, the Overall magnitude of ICCs was 74 (24.3%) (Figure 1).

### 3.2 Number of patients with intraoperative cardiac complication

**Figure 2:**
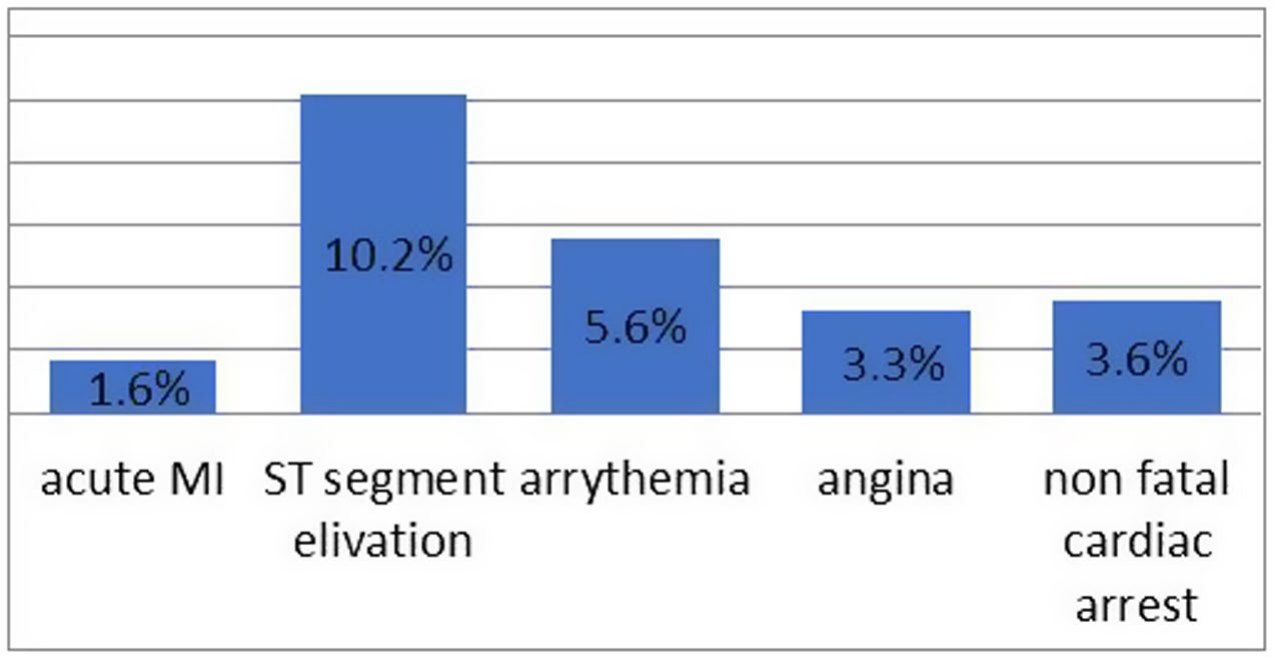
Number of patients with major adverse cardiac events (MACEs).

ICC complications occurred in 74 (24%) of the study population. Among patients who developed ICC, intraoperative ST abnormality on ECG was the major complication accounting for approximately30 (10.2%) (Figure 2).

### 3.3 Number of patients with ischemic complications

Among patients who developed ICC, 92 (33.3%) had ischemic complications; in addition, intraoperative MI was 46 (15.1%; Table 2).

**Table 2:** Number of patients with ischemic complications at selected SNNPR governmental hospitals, from March 2022 to August 2022.

ICCS	Percentage with ICC (=74)	Percentage without ICC (*n* = 230)
Ischemic complication	92 (33.3%)	212(69.7%)
Ischemia	10 (3.3%)	294(96.7%)
Angina	19(6.3%)	285(8 1%)
MI	46(15. 1)	258(84.9%)
Pathological Q wave	17(5.6%)	287(94.4%)

ICC, intraoperative cardiac complication; MI, myocardial infarction.

### 3.4 Preoperative factors associated with ICC

Output of binary logistic regression analysis finding: age, BMI, University of California and Los Angeles (UCL), ASA, history of hypertension, history of angina, and preoperative ECG abnormalities, as preoperative associated factors, are a potential candidate for the final model (with a *p*-value < 0.25). On the other hand, the Revised Cardiac Risk Index (RCRI), preoperative Hgb, history of HF, and preoperative creatinine did not show any association after binary logistic regression.

### 3.5 Intraoperative factors associated with ICC

Intraoperative-associated factors that were a potential candidate for the final model (with a *p*-value of <0.25) after binary logistic regression analysis included intraoperative hypotension, type of surgery, duration of anesthesia, intraoperative hypoxia, and intraoperative blood loss. In contrast, the type of anesthesia used, type of surgery, maintenance agents, and intraoperative blood transfusion were not candidates for the final model after binary logistic regression analysis.

### 3.6 Perioperative-associated factors of ICC with final model analysis

**Figure 3:**
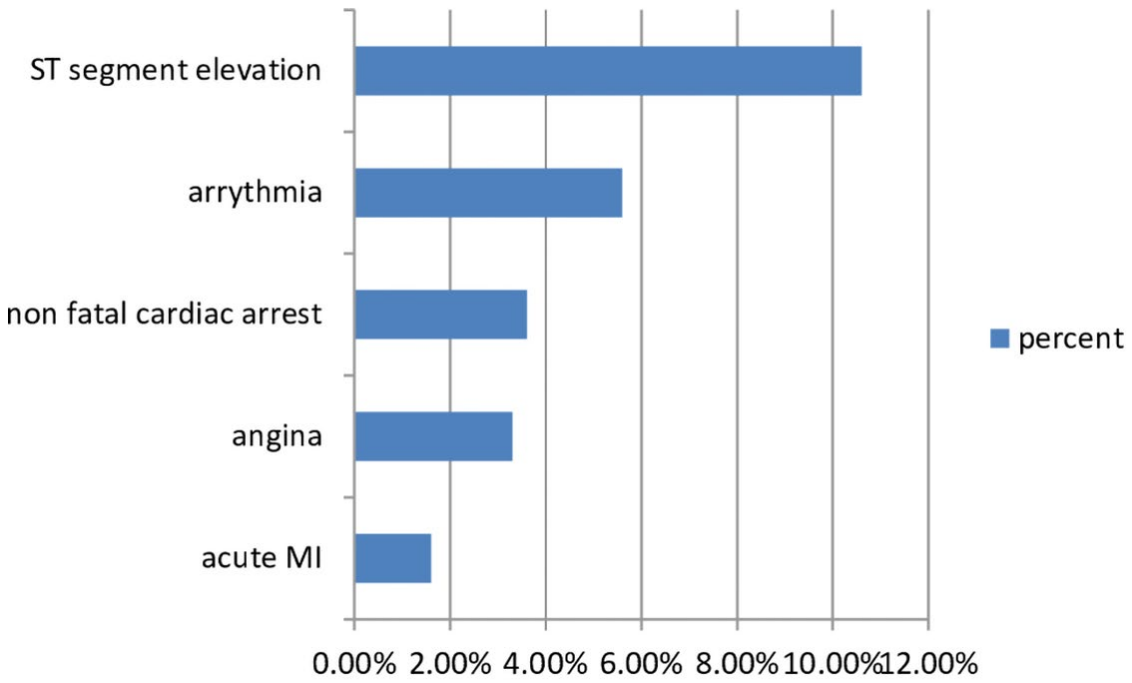
Number of patients with intraoperative cardiac complications.

Factors associated with ICC were analyzed using binary logistic regression, and variables with a *p*-value of <0.25 were used in the model for final analysis. Accordingly, the output of the multiple logistic regression model revealed six independent factors: Patients aged >85 years were six times more likely to develop intraoperative cardiac complications when compared with patients aged 85 years (AOR = 6.01, CI = 5.12–12.21). The result indicates that patients who had a preoperative history of hypertension were more than three times more likely to develop intraoperative cardiac complications than those patients who had no history of hypertension (AOR =3.424, CI =2.02–6.080). The study showed that ST-segment abnormality was two times more likely to develop intraoperative cardiac complications when compared with other ECG findings (AOR = 2.433, CI = 2.06–3.67) (Figure 3; Table 3). Among intraoperative factors, intraoperative hypotension was identified as the strongest preoperative associated factor of ICC with [AOR of 6.20, CI = 6.20(3.5–110.9)]. A patient under anesthesia for more than 3 h was two times more likely to develop intraoperative cardiac complication compared to those patients under anesthesia for less than 3 h (AOR =2.27, CI = 2.0.2–18.25). The most significant preoperative related factors of ICC were found to be patients who experienced intraoperative hypoxia, as they were three times more likely to experience intraoperative cardiac problems than those who did not (AOR = 3.598, CI = 2.07–6.23) (Table 3).

**Table 3:** Output of multiple logistic regression analysis showing independent predictors of ICC among surgical elective patients at governmental hospitals from March 2022 to August 2022.

Variables		ICC		COR (95% CI)	AOR (95% CI)	*P*-value
		Present	Absent			
Age	65–74	20 (6.6%)	13 (4.3%)	1	1	
	75–84	19 (6.2%)	199 (65.5%)	15.7(4.7–23.24)	4.87(3.7–8.3)	0.001
	>85	35 (11.5%)	18 (5.9%)	17.75(4.667.67)	6.01(5.12–12.21)^*^	0.001
BMI	<18.5	4 (1.3%)	5 (1.6%)	1	1	
	18.5–24.5	7 (2.3%)	194(63.8%)	16.80(1.526–18.4)	7.75(0.65–15.76)	0.72
	25–30	62 (20.4%)	10 (3.3%)	2.175(2.07–10.93)	0.24(0.14–1.68)	0.15
	>30	1 (0.3%)	21(6.9%)	16.8(1.53–18.93)	0.06(0.05–0.65)	0.03
ASA classification	II	26 (8.6%)	214(70.4%)	0.04 (0.02–0.08)	0.05(0.02–0. 122)	0.41
	III	48(15.79)	16(5.3%)	1	1	
UCLA classification	Intermediate	40(13.2%)	199(65.8%)	5.19(2.9–9.43)	7.97 (5.63–9.34)	0.02
	High	34(11.2%)	32(10.5%)	1	1	
Hx of hypertension	Yes	31(10%)	43(14%)	3.424(1.29–6.08)	3.42 (2.02–6.08)^**^	<0.001
	No	40(13.1%)	190(62.3)	1	1	
Smoking Hx	Yes	26(8.6%)	33(10.9%)	17.25(7.08–42.0)	15.81(6.44–38.8)	0.021
	No	48(15.8%)	223(73.4%)	1	1	
Hx of HF	Yes	18(5.9%)	56(18.4%)	1.677(0.88–3. 17)	1.25 (0.60–2.65)	0.526
	No	37(12.2%)	193(63.5%)	1	1	
Preoperative ECG	ST elevation	26(13.85)	32(17.7%)	0.137(0.03–0.64)	2.43(2.06–3.67)*	0.02
	VH	11 (4.7%)	45(24.9%)	0. 133(0.26–0.67)	2.36(0.294–4.97)	0.418
	BBB	11(6%)	19(10.5%)	0.396(0.087–1.80)	1	
Hx of angina	Yes	17(5.6%)	57(18.8%)	6.896(2.67–17.7)	1.88(0.47–7.49)	0.366
	No	10(3.3%)	220(72.4)	1	1	
Anesthesia duration	<3 h.	53(17.4%)	211(69.4%)	1	1	
	>3 h.	21(6.9%)	19(6.25%)	0.089(0.01–0.69)	2.27(2.0.2–18.25)*	0.001
Blood loss	<500 mL	36(11.8%)	106(34.9%)	1	1	
	>500 mL	126(41.4)	38(12.5%)	0.721(0.48–1.06)	2.498(0.21–5.64)	0.71
Type of anesthesia	GA	59(8.9%)	132(43%)	5.343(4.54–9.3)	5.298 (2.83_9.88)	0.041
	SA	98(32%)	15(4.9%)	1	1	
Intra-op hypotension	Yes	44(14.5%)	44(14.5%)	6.2(3.52–10.99)	6.20(3.5–110.9)**	<0.001
	No	30(9.6%)	186(61.2%)	1	1	
Intra-op hypoxia	Yes	33(10%)	41(13.4%)	2.809 (2.02–4.83)	3.59(2.07–6.23)**	<0.001
	No	130(42.7)	137(45%)	1	1	

BMI, body mass index; ASA, American Association of Anesthesiologists; UCLA, the University of California and Los Angeles; HF, heart failure; COR, crude odds ratio; AOR, adjusted odds ratio; CI, confidence interval; *n*, number; %, percent; SA, spinal anesthesia; *, significantly associated variables that are a candidate for final analysis model at *P*-value less than 0.05; Hx, history.

## 4 Discussion

One of the major challenges in the management of geriatric surgical patients is the prediction of intraoperative cardiac complications. In this study, the prevalence of intraoperative cardiac complications among elderly patients who underwent non-cardiac surgery was 24.3%.

Intraoperative ST abnormality on ECG was the major complication, accounting for approximately 30 (10.2%). In addition, 92 (33.3%) patients with ischemic complications and intraoperative MI accounted for 46 (15.1%). Age > 85 years, preoperative history of hypertension, ST-segment abnormality, intraoperative hypotension, patient under anesthesia for more than 3 h, and intraoperative hypoxia compared to the counterpart are the strongest perioperative factors associated with ICC. In most studies, the occurrence of ICC and intraoperative ischemic complications were more or less similar in frequency among geriatric populations (18, 23). This could be a result of interindividual differences in anesthetic management in terms of their preoperative assessment, intraoperative care, and management during surgery, which may also affect the severity of intraoperative cardiac problems.

The findings of this study are higher than the results of research conducted in university hospitals located in different regions of China (9.1%) and at Peking Union Medical College Hospital in China (19.4%) (14, 24). The findings of this study are also higher than the findings of the studies conducted in Europe, which were in the range of 0.5–3.5% ICC reported by various authors (25, 26). Poor surgical case selection, lack of progress in the preoperative management of cardiovascular risk factors and disease, and outdated surgical and anesthesia techniques used during surgery, including the inability to use minimally invasive surgical interventions that would shorten the duration of anesthesia, could all be contributing factors to the observed increase. Therefore, perioperative optimization and intraoperative best management together can reduce ICC.

In this study, age > 85 years was strongly associated with ICC, which agrees with previous studies conducted in Chinese in 2004 and 2017 (18, 27). Even though all subjects of this study were elderly, over 65 years of age, it was still associated factor of ICC. This might be because of age-related cardiovascular changes such as a decrease in number of cardiac myocytes, and the remaining myocytes undergo hypertrophy and a poor response to hemodynamic change, which can lead to ICC. Therefore, knowing physiological changes and avoiding intraoperative hemodynamic instability can help decrease ICC. However, the findings of current study disagree with the findings of the studies reported in most central and eastern European countries, including the USA (28, 29). In many central and eastern European countries, the mortality rates of geriatric patients have declined in those aged over 65 years. Significant variations exist and may be due to differences in the underlying disease, sociodemographic factors, and quality of care.

The present study indicates that preoperative ECG (ST abnormality) was positively associated with ICC. Similar to these studies, studies conducted in the Chinese population identified the significance of ST-segment abnormalities in addition to clinical characteristics as an independent risk factor for ICC (18, 30).

However, studies done in Asia and California’s San Francisco University reported ECG findings of LVH and BBB increased the odds of developing a subsequent intraoperative cardiac complication seven to nine times (31), which was not shown in this study. Similar controversy exists regarding the predictive value of ST-wave changes to ICC (17, 18). The study population (vascular verses general surgery), the Sokolow-Lyon verses Minnesota Code for LVH criteria, associated factors, and the age of patients enrolled may be reasons for variation from current study result. This finding suggests that evaluating preoperative ECG and intraoperative monitoring in geriatric patients undergoing non-cardiac surgery could be critical.

In this study, intraoperative hypoxia was positively associated with ICC. This might be because the mismatch between oxygen supply and demand and the catecholamine stress caused by pain, which increases the oxygen consumption of the heart, is the classic mechanism of ICC (12). The present finding is consistent with many previous studies (18, 24). This finding is also similar to the study that examined the electronic anesthetic records of 95,407 patients and discovered that the prevalence of intraoperative hypoxia was 6.8% (32). These findings indicate that a surprisingly high percentage of patients (10%) who had sustained hypoxemia during surgery developed ICC. However, the present finding disagrees with the finding of the study conducted at the University of California, San Francisco, which reported a low prevalence of hypoxia with a low association with ICC (30). The differences may be because the current study was conducted on participants with underlying different types of disease, whereas other studies were conducted only on patients with CVD.

There was a strong association between intraoperative hypotension and ICC found in this study, which can be explained by the higher risk of hypotension causing myocardial injury, myocardial infarction, and cardiogenic shock in patients undergoing non-cardiac surgery (33–35), which is consistent with the studies that provide evidence that hypotension is associated with a 36% increase in the risk of cardiovascular events, which causes decreases in both systemic vascular resistance and cardiac output, which results in cardiac complications (24, 36), but the findings of this study disagree with the findings of the study reported in China (18). This variation might be due to differences in the underlying disease and the quality of intraoperative management. Maintained preloading to the heart during surgery, anesthesia management, and provide adequate intravenous fluids can be satisfactory in most cases.

This study found that the duration of anesthesia (>3 h) was a significant factor associated with ICC. The justification could be that as time is prolonged, the increment of blood loss, anesthetic use, and hemodynamic instability can occur, which led the patient to have ICC. Several studies have hypothesized and reported associations between prolonged anesthesia time and cardiac complications (13, 37). However, the evidence for the duration of anesthesia and its influence on intraoperative cardiac complications remains poor, particularly in the surgical literature, which did not report it as an independent predictor of ICC (18, 30). The observed variation may be due to the differences in the advancement of surgical and anesthesia management in the study area, which significantly decrease the duration. Improving perioperative surgical and anesthesia management with available resources can minimize the duration of surgery.

This study also indicated stage 3 hypertension with regular treatment as associated factor of ICCs. Multiple studies have shown that not only is it safe to treat hypertension in geriatrics but also that it will decrease stroke, HF, myocardial infarction, and all other causes of mortality in non-cardiac surgery (25, 26). A study that enrolled 587 geriatric patients in America in the College of Cardiology and the Chinese population demonstrated that a history of hypertension requiring medication was a significant risk factor for developing cardiac complications (18, 38), but there are also legitimate concerns that tight BP control may increase the risk of hypotension in the geriatric population. Therefore, the combination of preoperative optimization and maintaining intraoperative hemodynamic stability can decrease ICC.

Several associated factors considered important by others were not show significance in our study, including a history of MI, gender, and classification of surgical risk (18). Furthermore, there was also no significant association seen between intra-operative blood loss of less than two units of blood and an ASA categorisation of greater than three (30). The reason for this difference could be that there were few patients with these diagnoses and most were successfully stabilized preoperatively, which could also be secondary to the difference in study population attributed to the optimization of patients before elective surgery and improvements in the surgical procedure. In general, the preoperative physical functional status according to RCRI is clinically very important/significant toward ICC, but in our study it is not statically significant.

## 5 Limitation of the study

Limited resources to print intraoperative ECG could make the interpretation more accurate.

## 6 Strength of the study

There was an adequate sample size within the planned period as this was a multi-center study.

## 7 Conclusion

This study showed that intraoperative cardiac complications are common among geriatric patients who undergo non-cardiac surgery at SNNPR governmental hospitals. The associated factors of intraoperative cardiac complications for this population included age > 85 years, ST-segment elevation, perioperative hypertension (stage 3 with regular treatment), duration of anesthesia >3 h, Intraoperative hypoxia, and intraoperative hypotension.

## 8 Recommendation

Pre-surgical evaluation and prevention of intraoperative hypoxia and hypotension may help to reduce the number of intraoperative cardiac complications.

Healthcare providers including the anesthetist should be aware of all possible factors and develop a strateg1y to minimize the complication.

## Data availability statement

The original contributions presented in the study are included in the article/supplementary material, further inquiries can be directed to the corresponding author.

## Ethics statement

The studies involving humans were approved by College of Health Science WSU Ethical Review Board. The studies were conducted in accordance with the local legislation and institutional requirements. Written informed consent for participation in this study was provided by the participants’ legal guardians/next of kin.

## Author contributions

AA: Conceptualization, Data curation, Formal analysis, Investigation, Methodology, Project administration, Supervision, Validation, Writing – original draft, Writing – review & editing. MT: Formal analysis, Supervision, Writing – review & editing. TT: Investigation, Methodology, Validation, Writing – review & editing. ADe: Investigation, Software, Writing – review & editing. EH: Data curation, Investigation, Software, Writing – review & editing. AS: Formal analysis, Investigation, Writing – review & editing. GD: Formal analysis, Investigation, Methodology, Software, Writing – review & editing. NG: Data curation, Formal analysis, Investigation, Software, Writing – review & editing. ADa: Formal analysis, Software, Supervision, Writing – review & editing. MS: Formal analysis, Investigation, Methodology, Software, Writing – review & editing.
